# Biallelic variants in *NOS3* and *GUCY1A3*, the two major genes of the nitric oxide pathway, cause moyamoya cerebral angiopathy

**DOI:** 10.1186/s40246-023-00471-x

**Published:** 2023-03-20

**Authors:** Stéphanie Guey, Dominique Hervé, Manoëlle Kossorotoff, Guillaume Ha, Chaker Aloui, Françoise Bergametti, Minh Arnould, Hind Guenou, Jessica Hadjadj, Fanny Dubois Teklali, Florence Riant, Jean-Luc Balligand, Georges Uzan, Bruno O. Villoutreix, Elisabeth Tournier-Lasserve

**Affiliations:** 1 Inserm UMR-S1141, Université Paris Cité, Paris, France; 2 Service de Neurologie, Centre de Référence des Maladies Vasculaires Rares du Cerveau et de L’Oeil, Hôpital Lariboisière, AP-HP, 75010 Paris, France; 3 Department of Pediatric Neurology, French Center for Pediatric Stroke, AP-HP, University Hospital Necker-Enfants Malades, Paris, France; 4 Inserm U1266, Paris, France; 5 INSERM, UMR-S-MD 1197, Hôpital Paul Brousse, Université d’Evry-Val-d’Essonne, Université Paris-Saclay, 94800 Villejuif, France; 6 Service de Génétique Moléculaire Neurovasculaire, Hôpitaux Lariboisière-Saint-Louis, AP-HP, 75010 Paris, France; 7 Department of Pediatrics, Grenoble University Hospital, 38000 Grenoble, France; 8 Pole of Pharmacology and Therapeutics (FATH), Institute of Experimental and Clinical Research (IREC), Cliniques Universitaires Saint-Luc, Université Catholique de Louvain (UCLouvain), Brussels, Belgium

**Keywords:** Moyamoya, Stroke, Nitric oxide, Soluble guanylate cyclase, Nitric oxide synthase

## Abstract

**Background:**

Moyamoya angiopathy (MMA) is a rare cerebrovascular condition leading to stroke. Mutations in 15 genes have been identified in Mendelian forms of MMA, but they explain only a very small proportion of cases. Our aim was to investigate the genetic basis of MMA in consanguineous patients having unaffected parents in order to identify genes involved in autosomal recessive MMA.

**Methods:**

Exome sequencing (ES) was performed in 6 consecutive consanguineous probands having MMA of unknown etiology. Functional consequences of variants were assessed using western blot and protein 3D structure analyses.

**Results:**

Causative homozygous variants of *NOS3*, the gene encoding the endothelial nitric oxide synthase (eNOS), and *GUCY1A3*, the gene encoding the alpha1 subunit of the soluble guanylate cyclase (sGC) which is the major nitric oxide (NO) receptor in the vascular wall, were identified in 3 of the 6 probands. One *NOS3* variant (c.1502 + 1G > C) involves a splice donor site causing a premature termination codon and leads to a total lack of eNOS in endothelial progenitor cells of the affected proband. The other *NOS3* variant (c.1942 T > C) is a missense variant located into the flavodoxine reductase domain; it is predicted to be destabilizing and shown to be associated with a reduction of eNOS expression. The *GUCY1A3* missense variant (c.1778G > A), located in the catalytic domain of the sGC, is predicted to disrupt the tridimensional structure of this domain and to lead to a loss of function of the enzyme. Both *NOS3* mutated probands suffered from an infant-onset and severe MMA associated with posterior cerebral artery steno-occlusive lesions. The *GUCY1A3* mutated proband presented an adult-onset MMA associated with an early-onset arterial hypertension and a stenosis of the superior mesenteric artery. None of the 3 probands had achalasia.

**Conclusions:**

We show for the first time that biallelic loss of function variants in *NOS3* is responsible for MMA and that mutations in *NOS3* and *GUCY1A3* are causing fifty per cent of MMA in consanguineous patients. These data pinpoint the essential role of the NO pathway in MMA pathophysiology.

**Supplementary Information:**

The online version contains supplementary material available at 10.1186/s40246-023-00471-x.

## Introduction

Moyamoya angiopathy (MMA) is characterized by a progressive stenosis of the intracranial internal carotid arteries (ICA) and their bifurcation proximal branches, associated with the development of thin collateral vessels at the base of the brain. Its pathophysiology is still unknown [1]. Ten to fifteen percent of MMA are familial cases, in East Asian countries as in the West [2, 3]. Molecular data published during the last decade have established the role of genetic factors in MMA pathogenesis. These genetic factors include low penetrance susceptibility variants in the *RNF213* gene and highly penetrant variants in 15 other genes that are involved in Mendelian MMA syndromes [4]. MMA is indeed a highly heterogeneous condition, and MMA patterns of inheritance are variable. Indeed, MMA may be encountered in autosomal dominant conditions (e.g. RASopathies such as type 1 neurofibromatosis (MIM 162200) or Noonan syndromes (MIM 163950), and Alagille syndrome (MIM 118450), autosomal recessive one (e.g. in Majewski (MIM 210720) or Seckel syndrome (MIM 210600), sickle cell disease (MIM 603903), Aicardi-Goutieres syndrome due to *SAMHD1* mutations (MIM 225750), and X-linked conditions (e.g. MMA caused by *BRCC3-MTCP1* deletions, MYMY4 (MIM 300845). Numerous cases are also isolated cases. Genes that have been involved in MMA belong to various signaling pathways. The identification by our research team in 2014 of 3 families showing a syndromic MMA resulting from biallelic disruptive variants of *GUCY1A3*, MYMY6 (MIM 615750) suggested a central role of nitric oxide (NO) in pathophysiology of MMA [5]. However, no other gene belonging to the NO pathway has been shown so far to be mutated in MMA patients.

A Mendelian inheritance must be suspected in cases of syndromic MMA (i.e., MMA associated with other neurological or extraneurological symptoms) or when the familial context suggests a mendelian heritability (aggregation of intrafamilial cases, or consanguinity). However, despite an extensive screening of known MMA genes, a causative mutation is nowadays identified in a very limited proportion of these cases, strongly suggesting that additional MMA genes remain to be identified. Herein we conducted an exome sequencing (ES) analysis of 6 consanguineous probands aiming to identify new autosomal recessive forms of MMA.

## Methods

### MMA probands

The 6 probands analyzed in the present study were part of a cohort of 126 consecutive unrelated probands having a MMA of unknown etiology. These 126 MMA probands had been referred to the Department of Genetics at Lariboisière Hospital (Paris, France), to the French Center for Pediatric Stroke, to the French National Center for Rare Vascular Diseases of the Brain and the Eye (CERVCO), and to the Department of Neurology at the Alfried Krupp Hospital (Essen, Germany). This MMA cohort included a total of 6 unrelated consanguineous cases who were the focus of this study designed to identify genes involved in autosomal recessive MMA. All these consanguineous probands were single cases (Additional file 1: Figure S1). In each proband, a check-up aiming to exclude a secondary cause of MMA had been carried out before the inclusion. This check-up comprised an electrophoresis of hemoglobin, the search for history of head or neck irradiation, meningitis, achalasia, or Raynaud phenomenon, the search for dysmorphic features, short stature, or puberty delay, and a biological check-up including auto-immune, thyroid, and coagulation assessment, and a research of mutation or deletion in BRCC3-MTCP1 in male probands. [4] Probands and family members provided their written informed consent for genetic analysis as requested by the French law.

### Exome sequencing and exome data analysis

Genomic DNA of the probands was isolated from peripheral blood leukocytes using the Wizard Genomic DNA Purification Kit. ES was performed at IntegraGen platform (Evry, France) using the SureSelect Human All Exon V5 Clinical Research Exome kit of Agilent and were sequenced with an Illumina HiSeq2000 (paired end, 75 bp reads). Mapping and variant calling were performed with the CASAVA pipeline provided by Illumina. Reads were mapped to the GRCh37 build using ELAND software. Single-nucleotide substitutions and small insertion-deletion (indel) variants were annotated with an Integragen bioinformatics pipeline.

Based on the knowledge of a consanguinity in six probands, we interrogated their exome data under the assumption that the causative variant might be a rare homozygous variant. Given the very high genetic heterogeneity observed in MMA, we choose to analyze separately ES data of each consanguineous proband.

To limit the number of false positives, we considered only variants with a Q Phred quality score ≥ 30 and a coverage ≥ 8X. We restricted our analysis to nonsense, missense substitutions, mutations in canonical splice-sites and small insertions-deletions located in coding regions. The public databases dbSNP 144, 1000 Genomes Phase 3, Exome Sequencing Project (ESP6500SIV2), ExAC (0.3) and gnomAD (v3.1.2) were screened for each coding variant detected. Variants with a minor allele frequency (MAF) exceeding 1% in public databases were considered as polymorphisms and were excluded. Variants found in more than 1% of controls belonging to an internal Integragen database were also excluded (Filter 1). Remaining variants were excluded if their allele frequency exceeded 1% in the ExAC ethnic subgroup fitting with the proband’s ethnicity (Filter 2). Pathogenicity of missense variants was predicted using the Mutation Taster, SIFT and Polyphen 2 (HumVar) web servers. Missense variants were excluded if they were predicted benign by more than one out of the three software (or if they were predicted benign by at least one software when the prediction was available for two software only) (Filter 3). Combined Annotation Dependent Depletion (CADD) pathogenicity scores were calculated for the remaining candidate variants.

### Analysis of the functional consequences of the variants identified in NOS3 and GUCY1A3

#### Characterization of the mRNA splicing effect of the c.1502 + 1G > C variant of NOS3 identified in proband M084

Total RNA was extracted from whole blood circulating cells of M084 using the PAXgene Blood RNA kit (PreAnalytiX), and was retrotranscribed using a Maxima synthesis kit (Thermo Scientific). cDNA was sequenced by the Sanger method.

#### Characterization of the consequences at the protein level of the NOS3 c.1502 + 1G > C splice variant

*NOS3* encodes for the endothelial NOS (eNOS), whose expression is highly restricted to endothelial cells. However, a small fraction of circulating endothelial progenitor cells (EPC) also expresses the eNOS protein, providing a non-invasive access to eNOS expressing cells.

Circulating EPC were extracted from 80 ml of blood of M084 as previously described in [6]. Briefly, blood mononuclear cells were isolated from a Ficoll gradient and plated into collagen I (50 µg/ml) precoated 6- or 12- well plates in EGM2 medium (Lonza). The next day, cells were washed with 1X PBS and non-adherent cells were discarded daily for 7 days when the medium was changed. Thereafter, the medium was changed every other day.

EPC protein lysates were obtained using a lysis buffer (62.5 mM Tris pH6.8, glycerol 10%, SDS 2%) at room temperature. The same experimental protocol was conducted on EPC generated from 3 healthy female control. Lung protein lysates obtained from C57bl/6 wild-type mice and *NOS3*-/- knock-out littermates were used as positive and negative murine controls. Proteins were extracted using RIPA buffer supplemented with protease inhibitors. Proteins were quantified using BCA protein Assay kit (Pierce). 15 µg of human EPC lysates (M084 and control) and 30 µg of murine lung lysates were loaded on 3–8% Tris–Acetate gel. Proteins were transferred to nitrocellulose 0.45 µm membranes and incubated with primary antibodies directed against the AA 2–160 of the human eNOS (1:500, Santa Cruz monoclonal B-5) and against ß-actin (1:10,000, Sigma monoclonal AC-15). The secondary antibody used was a polyclonal goat anti-mouse peroxydase (1:3000, Cell Signaling 7076).

#### Characterization of the functional consequences of the NOS3 c.1942 T > C/p.C648R missense variant identified in proband M035

As we could not get EPC from M035, who was severely affected, we used Embryonic Kidney 293 (HEK293) cells transfected with a mutated construct to assess the stability of the mutated eNOS protein. Briefly, the full-length cDNA of human *NOS3* (plasmid HsCD00399526, Harvard Medical School) was cloned into the expression vector pCDNA3.1, using the Seamless PLUS cloning and assembly kit (Invitrogen). The c.1942 T > C variant was introduced into the cDNA using primers available upon request. The wild-type and mutated plasmids were then transfected into HEK293 cell lines, using the Dharmafect duo according to the manufacturer’s instructions (Dharmacon). Expression vector pCDNA3.1 containing the wild-type cDNA was also transfected into HEK293 cell lines using the same protocol. Cells were incubated during 48 h before RNA and protein extraction. Proteins were extracted using RIPA buffer, and quantified using BCA protein Assay kit. Expression of eNOS was assessed by western-blot after loading 10 µg of cell lysates, using the same protocol as described above. Total RNA was extracted using the NucleoSpin RNA Plus kit (Macherey–Nagel), and was retrotranscribed into cDNA using Maxima synthesis kit with DNAse. Comparative *NOS3* mRNA amount was assessed by RT-qPCR using a Light Cycler 480 RT-qPCR system (Roche), SYBR Green kit and specific primers (available upon request). Expression levels were normalized with the TBP and G6PD housekeeping genes.

### 3D structural analysis of the effects of *NOS3* and *GUCY1A3* missense variants

Methodological details are provided in the Additional file 2.

Briefly, the *NOS3* mutated residue, C648, is located in the reductase module for which no experimental 3D structure is available. However, an accurate homology model for the reductase module of human eNOS could be built (via the SWISS-MODEL web-server) using as structural template the corresponding domain of the rat neuronal nitric oxide synthase. The theoretical 3D model of the full-length human eNOS protein was downloaded from the AlphaFold Protein Structure Database powered by the AlphaFold2 structural prediction engine developed by DeepMind. The 3D experimental structures and models were then analyzed using various approaches including Chimera and PyMol.

With regard to *GUCY1A3*, the cryo-electron microscopy structures of the human sGCα1β1 heterodimer in different functional states were available and could be downloaded from the RCSB Protein Data Bank. The bent conformation is considered to represent the inactive state while the NO-activated state is in an extended conformation. The mutated residue, R593, is located in the catalytic module of the alpha1 subunit of the human sGCα1β1 heterodimer. As above, the two 3D structures were analyzed with various computational approaches.

## Results

Genealogical trees of the six consanguineous sporadic cases are shown in Additional file 1: Figure S1. Four of the consanguineous probands originated from Maghreb (M030, M035, M084 and M101) and two were of Middle-East origin (M038 and M116). All but one were females. Four probands had their first symptoms during infancy or adolescence, including two probands with a very early onset during their first year of life (M035 and M084).

### Identification in three of the six probands of rare homozygous coding variants in *NOS3* and* GUCY1A3*

Thirty-four homozygous rare coding non-synonymous variants, belonging to 33 distinct genes, were identified in the 6 consanguineous probands (Table 1 and Additional file 3: Table S1).

**Table 1:** ES filtering data obtained for the six consanguineous MMA probands

Proband ID	Number of homozygous candidate variants			*V*ariants in *NOS3* (NM_000603) and *GUCY1A3* (NM_000856.5)
	Filter 1	Filter 2	Filter 3	
M030	8	6	3	0
M035	21	18	11	1 homozygous missense substitution in NOS3 (c.1942 T > C, p.C648R)
M038	35	29	13	1 homozygous missense substitution in GUCY1A3 (c.1778G > A, p.R593H)
M084	20	11	1	1 homozygous splice-site substitution in NOS3 (c.1502 + 1G > C)
M101	7	4	2	0
M116	11	11	4	0

Filter 1: homozygous nonsense, stop-loss, canonical splice-sites, indel and missense variants, coverage ≥ 8X, Q Phred score ≥ 30, global Minor Allele Frequency (MAF) ≤ 1%

Filter 2: MAF ≤ 1% in the ExAC ethnic subgroup fitting with the proband’s ethnicity

Filter 3: exclusion of missense variants predicted as benign by > 1/3 in-silico software (or by ≥ ½ if prediction was available for two software only)

**Fig. 1:**
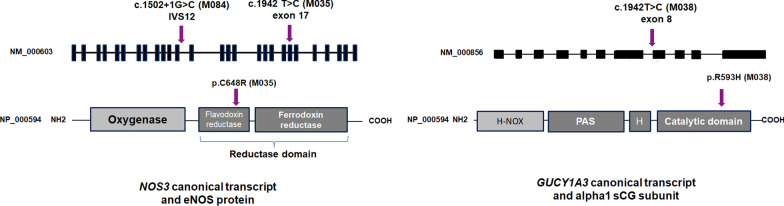
Schematic representation of the *NOS3* and *GUCY1A3* variant identified in M084, M035 and MP038 probands. Upper panels: NM_000603 and NM_000856 transcripts are the canonical transcripts for *NOS3* and *GUCY1A3* respectively, and are the ones that are referred in the present paper. Three shorter *NOS3* transcripts are referenced in Refseq (NM_001160109, NM_001160110 et NM_001160111), and encode for shorter protein isoforms that have been demonstrated to be non-functional [7]. Seventeen additional *GUCY1A3* transcripts are referenced in Refseq. Lower panels: Representation of the human eNOS (NP_000594) and the alpha subunit of sGC (NP_000847), that are referred in the present paper [8, 9]. The variants identified in *NOS3* and *GUCY1A3* in the present article are positioned on their respective transcript and protein. Legend: H-NOX: heme-NO/O_2_–binding domain; PAS: Per/Arnt/Sim domain, H: helical domain.

Variants of *NOS3*, the gene which encodes eNOS, were identified in M035 and M084 probands. It was the only gene to be mutated in more than one of the 6 consanguineous proband analyzed. One of these variants, the c. 1502 + 1G > C substitution identified in M084, was a disruptive variant. The other one, a c.1942 T > C substitution identified in M035, was a missense variant leading to the replacement of the cysteine 648 by an arginine (p.C648R) in the flavodoxine reductase domain of the protein (Fig. 1).

*GUCY1A3*, the gene that encodes a subunit of the sGC enzyme, the major NO receptor in the vascular wall, was found to be mutated at homozygous state in M038 proband. This c.1778G > A substitution (rs370478508) is a missense variant leading to the replacement of arginine 593 by histidine in the catalytic domain of the protein.

All three variants were confirmed by Sanger sequencing.

CADD scores for these three variants are respectively 27, 24 and 35. The two *NOS3* variants are absent from ExAC and GnomAD (v3.1.2) public databases and from the Greater Middle East (GME) database (http://igm.ucsd.edu/gme/data-browser.php), that lists variants identified in around 2500 Mediterranean and Middle East controls. Of note, interrogation of the GnomAD v3.1.2 public database found no homozygous *NOS3* disruptive variant carrier (i.e., nonsense, splice-site and frameshift insertions or deletions) after removing low quality variants. Cumulative Allele Frequency for heterozygous *NOS3* disruptive variants in GnomAD is near of 1/3000, establishing the rarity of such loss-of-function (LOF) variants in the *NOS3* gene. The *GUCY1A3* rs370478508 variant was absent from ExAC and present in a heterozygous state in only three carriers (2 African/African American and 1 European non-Finnish) in GnomAD (v3.1.2), with an allele frequency of 2 X10^−5^. It was absent from the GME database.

In the 6 consanguineous probands analyzed, ES data revealed no additional candidate variant neither in *RNF213* nor in genes known to be involved in Mendelian forms of MMA.

### Functional consequences of the *NOS3* c.1502 + 1G > C splice variant

**Fig. 2:**
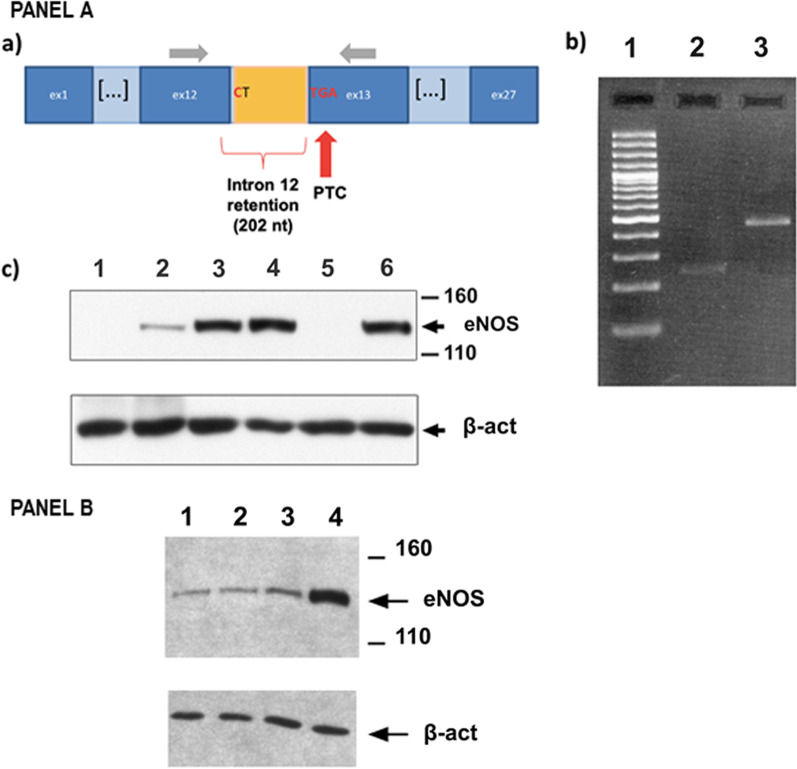
The two variants of *NOS3* detected in M035 and M084 probands are loss-of-function mutations. Panel **A**: The c.1502 + 1G > C splice-site variant identified in M084 causes a total loss of eNOS protein. (**a**) The c.1502 + 1G > C variant disrupts the canonical donor GT splice-site and causes intron 12 retention into the mRNA, resulting in a frameshift and in a premature termination codon in exon 13; Light grey arrows represent the primers used for cDNA amplification. *Abbreviations: nt* = *nucleotides; PTC* = *premature termination codon.* (**b**) Agarose gel electrophoresis migration of cDNA PCR amplicons from control (lane 2) and M084 (lane 3) using the primers shown in figure A (sequences provided on request). Lane 1: 100 base pairs ladder. The size of the amplicons obtained for M084 cDNA is about 200 nucleotides longer that those got from wild-type cDNA. Sequencing of amplicons showed a 202 nucleotides insert corresponding to the intron 12 retention into the mRNA. Detection of the mutated mRNA in M084 blood cells suggests that this mutant mRNA is spared by nonsense mRNA-mediated decay. (**c**) Western-blot performed on lysates form EPC derived from M084 proband and controls. Labelling with the monoclonal B-5 antibody directed against the N-terminal part of human and murine eNOS (Santa Cruz) showed a total loss of expression of eNOS in M084 proband (absence of signal on lane 5). Lane 1: NOS3 -/- knock-out mouse. Lane 2: wild-type mouse. Lane 3: EPC from healthy control 1. Lane 4: EPC from healthy control 2. Lane 5: EPC from M084 proband. Lane 6: EPC from healthy control 3. Panel **B**: The mutated p.C648R eNOS protein (c.1942 T > C variant) is unstable. HEK-293 cells were transfected with vectors containing the WT and mutated M035 full-length *NOS3* cDNA. Western-blot performed on lysates from transfected cells showed a strong reduction of eNOS protein amount in three independent clones (lanes 1–3) in comparison to the clone transfected with the wild-type cDNA (lane 4). The primary antibody used is the monoclonal B-5 (Santa-Cruz). Lane 1: c.1942 T > C mutated overexpression clone 1. Lane 2: c.1942 T > C mutated overexpression clone 2. Lane 3: c.1942 T > C mutated overexpression clone 3. Lane 4: wild-type overexpression clone

The c.1502 + 1G > C variant (NM_000603) identified in *NOS3* in a homozygous state in M084 was predicted to disrupt a canonical splice donor at RNA level (r.spl). Analysis of the cDNA prepared with RNA extracted from circulating blood cells of M084 showed that the c.1502 + 1G > C splice-site variant caused an intron 12 retention and led to a premature termination codon in exon 13 (Figs. 1; 2 Panel A, a et b; Additional file 4: Figure S2 Panel A.). This change in the open reading frame led to a truncated predicted protein (p.Ala502Trpfs*71).

Western-blot performed with EPC protein lysates from M084 showed that the c.1502 + 1G > C leads to a total lack of the mutant eNOS protein in circulating endothelial progenitor cells. The use of a monoclonal antibody directed against the N-terminal part of the protein allowed to exclude the expression of a truncated protein (Fig. 2 Panel A, c).

### Functional consequences of the *NOS3* c.1942 T > C (p.C648R) missense variant

The c.1942 T > C substitution of *NOS3* identified in a homozygous state in M035 leads to the p.C648R missense variant; this variant is located in the flavodoxine reductase domain of the protein (Fig. 1; Additional file 4: Figure S2 Panel B). As we could not get EPC from M035, we transfected HEK293 cells with a c.1942 T > C mutant cDNA in order to explore the functional consequences of this variant on eNOS stability. Western-blot showed a clear decrease in the eNOS protein amount detected in mutant HEK293 transfected cells (Fig. 2 Panel B), contrasting with a conserved mRNA expression in qRT-PCR experiments (Additional file 5: Table S2). Altogether, these data strongly suggest that the p.C648R mutated protein is unstable.

### Structural analysis of the p.C648R mutated eNOS

**Fig. 3:**
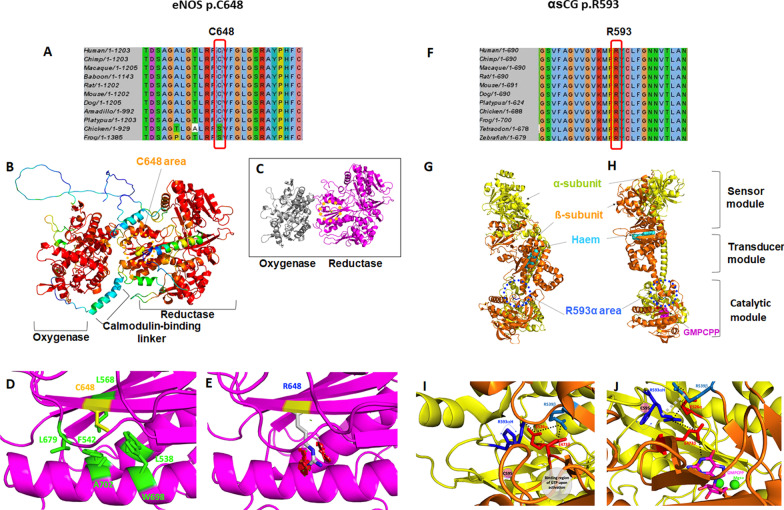
Structural modeling and in-silico analysis of the effect of missense variants identified in *NOS3* and *GUCY1A3*. **A** and **F**: Multiple Sequence Alignments (MSA) showing that the residues C648 of eNOS and R593 of alpha sCG are evolutionary conserved residues. **B** and **C**: 3D-structures of the wild type eNOS protein (experimental and 3D models). The C648 residue is located in the reductase domain of the protein. **D** and **E**: A zoom in the region of the C648 residue is presented in D (wild-type protein) and E (mutated p.C648R protein). The substitution p.C648R is predicted to destabilize the 3D-structure of the domain (DUET prediction ΔΔG = − 1.0 kcal/mol). There is no space to accommodate the larger and potentially positively charged arginine side chain in the region of the protein (steric clashes are represented by red and green cylinders).**G** and **H**: 3D-structures of the wild type sCG protein in an inactive (G) and NO-activated (H) states. sCG is a heterodimer composed of an α-subunit and a β-subunit. The R593 residue mutated in M038 is carried by the α-subunit and is located in the catalytic domain of sCG protein. **I** and **J**: A zoom in the region of the R593 residue is presented in I (wild-type inactive state) and J (wild-type NO-activated state). R593 takes part in a salt-bridge network that involves the evolutionary conserved residues E526 (α -subunit), R539 and E473 (β-subunit). E473 interacts with the GTP substrate when the sCG is activated (GMPCPP: GTP binding pocket). The substitution p.R593H is predicted to destabilize the 3D-structure of the inactive (ΔΔ G = -1.5 kcal/mol) and active forms (ΔΔG = − 1.60 kcal/mol) of sGC, through perturbation of non-covalent interactions in the catalytic domain, and negatively impact the formation of the GTP binding pocket

A multiple sequence alignment in the region of the mutation is shown in Fig. 3A, an overview of the full-length human eNOS AlphaFold2 model is presented in Fig. 3B while in the insert 3 C, the experimental structures of the human oxygenase module and of the predicted human reductase module based on the experimental structure of the rat protein are shown with the same orientation. A zoom in the region of residue C648 is presented in Fig. 3D. The exact orientation of the different domains is still not fully known but residue C648 can be analyzed as it is not directly at the interface with the other domains. C648 is located in the reductase module, in the middle of a β-strand, it is strictly conserved in the sequences (or replaced by a small Serine amino acid that has about the same volume and related properties although the polarity of the oxygen and sulfur atoms are different with an enhanced ability for Serine to form hydrogen bonds, Fig. 3A) and is fully buried. Its relative per-residue solvent accessible surface area (rSASA), for the side chain atoms was computed to be 0% [10]. This residue is in a tightly packed area and surrounded by hydrophobic and aromatic side chains. There is no space to accommodate a long and potentially positively charged arginine residue in this region of the protein. Independently of the rotamer selected, when replacing the cysteine by an arginine residue, severe clashes were noticed that could not be fixed by energy minimization (Fig. 3E). This substitution should thus induce local structural changes and is expected to be destabilizing given the environment and the type of amino acid substitution (i.e., small to large and potentially positively charged at least during some steps of the folding process). Stability predictions (computed with DUET) indicate that the p.C648R substitution should be destabilizing (ΔΔ G = − 1.0 kcal/mol).

These data strongly suggest that the p.C648R substitution is expected to be destabilizing and to perturb proper folding of this region of the protein. These data, in addition with the instability of the mutated eNOS detected on western blotting experiments, strongly suggest that this p.C648R variant is a loss of function variant.

### Structural analysis of the mutated p.R593H sGC alpha1 subunit

The cryo-electron microscopy structures of the human sGCα1β1 heterodimer are available for both the inactive state and the NO-activated state.

The 3D structure of the inactive sGC heterodimer is shown in Fig. 3 G while a close-up view in the area of R593 is presented in Fig. 3I. R593 is located on the α -subunit, in a loop structure and in the catalytic module. It is strictly conserved in the MSA (Fig. 3F) and is buried in the 3D structure; its rSASA was computed to be 13%. R593 is involved in a buried salt-bridge network involving E526 from the α -subunit (strictly conserved, located in a loop structure, rSASA = 28%) which also forms a salt-bridge with R539 from the β-subunit (strictly conserved, located in the N-terminal region of a β-strand, rSASA = 9%), that also forms a salt-bridge with E473 from the β-subunit (strictly conserved, located in a loop, rSASA = 14%). Of importance, the E473 side chain has polar interactions with the GTP substrate once the protein is activated (see below). Interactive amino-acid substitution of R593 α to histidine suggests that the newly introduced side chain could either significantly clashes into E526 α inducing local structural changes and destabilize the salt-bridge network or, alternatively, if some other rotamers are selected, positions the H side chain away from E526α, again destroying part of the salt-bridge network. According to the DUET stability computation, the p.R593H is predicted to be destabilizing (ΔΔG = − 1.50 kcal/mol).

The NO-activated structure is shown in Fig. 3 H and the region of R593 on the α-subunit is seen in Fig. 3J. Some important structural changes are noticed as compared to the inactive form and in the region of R593. For instance, in that region, some residues located in loop structures in the inactive state are now in a β-strand or the opposite and many residues in this region become even more shielded from the solvent. The salt-bridge network discussed above is also significantly modified while the binding pocket for the GTP substrate and cofactor Mg +  + ions is now fully formed. In the NO-activated form, R593 (α-subunit) is located in a loop structure and fully buried (rSASA = 2%). It has favorable hydrophobic-aromatic contacts with part of the side chain of R539 (β-subunit, now located in a loop, rSASA = 3%) and is part of a buried salt-bridge network involving both E526 (α-subunit, located in a loop, rSASA = 11%) and E473 (β-subunit, now located in a β-strand, rSASA = 12%). R539 (β-subunit) forms a salt-bridge with E526 (α-subunit) and E473 (β-subunit) has polar contacts with the GTP substrate. Assuming that an activated form of the mutant protein could still be formed, a histidine at position 593 should perturb the hydrophobic-aromatic-electrostatic interactions as compared to arginine independently of the exact position of the H side chain. According to the DUET stability prediction, the p.R593H in the activated form is predicted to be destabilizing (ΔΔG = − 1.60 kcal/mol).

Altogether, these data strongly suggest that the p.R593H substitution impacts the 3D structure in this region, the dynamics of the protein and its stability and thus affect the flow of information along the transducer module between the sensor module and the catalytic module.

### Clinical description of the probands harboring variants in *NOS3* and *GUCY1A3*

**Fig. 4:**
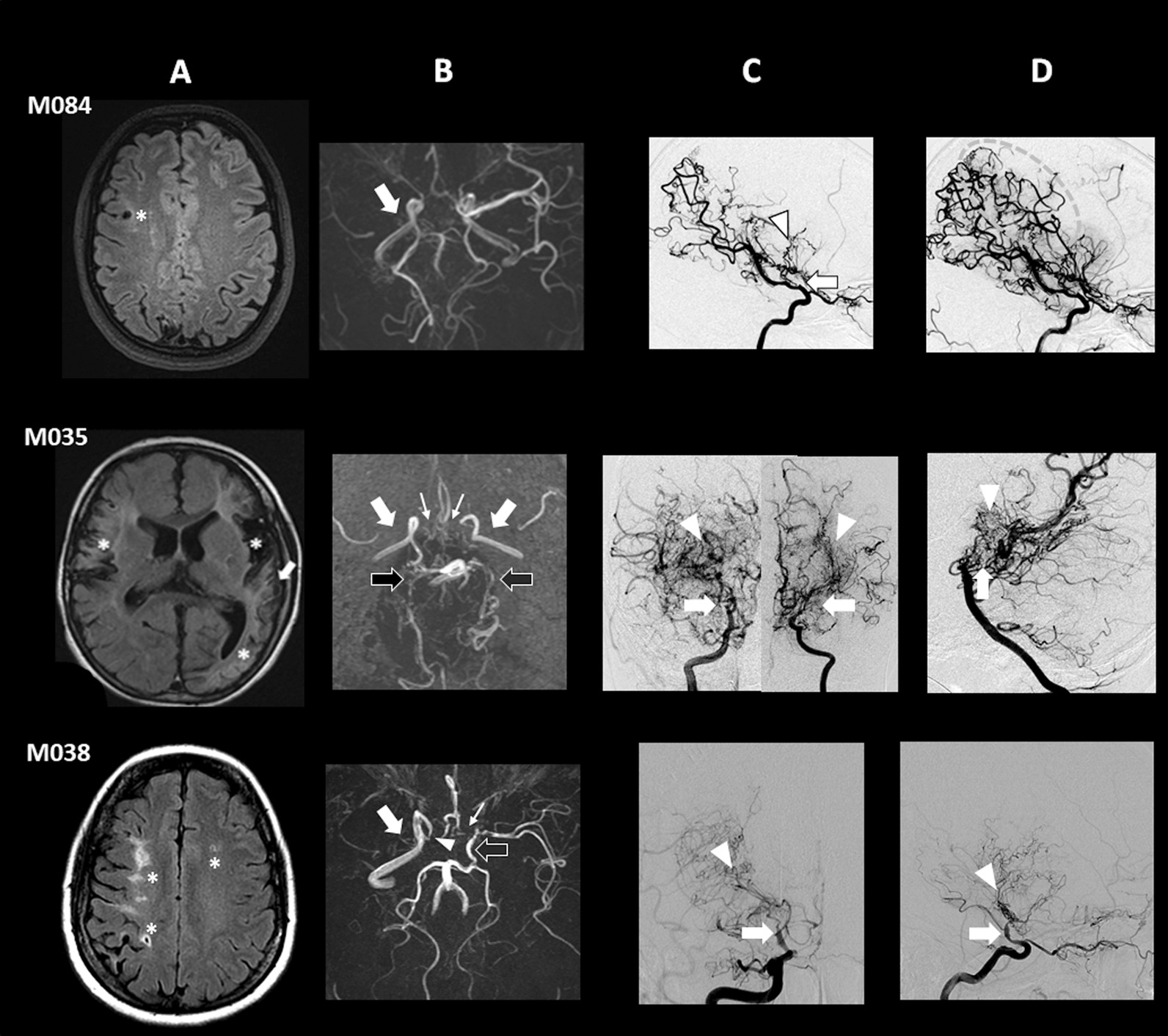
Imaging features of M035, M084 and M038 probands. **A**: MRI Fluid-attenuated inversion recovery (FLAIR) images. **B**: 3D Time-Of-Flight MR angiography (MRA). **C** and **D**: Digital subtracted conventional angiography. **M084 proband:** **A**: old cortical ischemic lesion (star) in the right MCA territory. **B**: Absence of right MCA (arrow). **C** (profile view, early contrast opacification time): Occlusion of the terminal right ICA downstream to the origin of posterior communicating artery (arrow) associated with bilateral deep collateral vascular network (arrowhead). **D** (profile view, late contrast opacification time): Note the presence of leptomeningeal collaterals from distal branches of the fetal right PCA participating in the blood supply of right MCA territory (dotted circle). **M035 proband:** **A**: Ischemic lesions (stars) in the right and left MCA territories; arterial hyperintensities (arrows) suggestive of Ivy sign. **B**: absence of the terminal segment of both ICAs and of the proximal segment of both MCAs (white arrows), ACAs (thin arrows) and PCAs (black arrows). **C** (frontal view): Steno-occlusive changes of the terminal ICAs bifurcation (arrows) associated with moyamoya vessels (arrowheads). **D** (profile view): Occlusion of both PCAs in their proximal segment (arrow), also associated with a collateral vascular network (arrowhead). **M038 proband:** **A**: Ischemic lesions (stars) in the right and left MCA territories. **B**: stenosis of the terminal part of right ICA (arrowhead), occlusion of the proximal segment of right MCA (arrow), and of left ACA (thin arrow). Terminal part of left ICA and proximal part of left MCA are fed by the left posterior communicating artery (black arrow). **C** (frontal view) and **D** (profile view): Multistage stenosis of the terminal part of right ICAs (arrows) associated with moyamoya vessels (arrowheads)

Proband M084 is a 31-year-old female born from healthy consanguineous parents (first cousins) originating from Morocco. Her familial and prenatal history were unremarkable. She had normal psychomotor development and graduated from university. From 9 months to 9 years old, she had repeated episodes of transient left hemiparesis especially during hyperventilation when crying. At 8 years of age, cerebral Magnetic Resonance Imaging (MRI) showed two old cortical infarcts in the right posterior cerebral artery (PCA) and middle cerebral artery (MCA) territories. A conventional angiography revealed an occlusion of the right terminal ICA bifurcation, a tight stenosis of the P1-P2 segment of the left PCA, and a long stenosis of the left anterior cerebral artery (ACA). A fine vascular network was observed close to the occluded arteries, thus confirming the diagnosis of MMA (Fig. 4). No abnormality was detected on trans-thoracic echocardiography and aorta CT angiography, and blood pressure was normal. A low-dose aspirin treatment was introduced. At 12 years of age, bilateral ocular hypertension, thin corneas, myopia, and a left homonymous hemianopia related to the posterior cerebral infarct were detected during a routine ophthalmological examination. The diagnosis of juvenile glaucoma was confirmed. After failure of medical treatment, a filtration surgery was finally performed at the age of 14 (both eyes) and 17 (left eye) years old, and the patient was subsequently treated with local ophthalmologic treatments. Thereafter, she remained stable from a neurological and ophthalmological point of view.

Proband M035 was the third child of consanguineous parents (first cousins) originating from Tunisia. No pathological condition was reported in his two older brothers. Prenatal history was notable for intra-uterine growth retardation diagnosed at 27 weeks of gestation and premature delivery threats requiring steroid treatment. He was born late preterm (34 weeks of gestation) and delivery required caesarean section due to abnormal fetal heart rate. Birth weight was 1615 g (5th percentile), birth length 40.5 cm (3rd percentile) and occipito-frontal circumference 29.5 cm (20^th^ percentile). Post-natal infancy was remarkable for neonatal hypoglycemia, short stature (< 3^rd^ percentile) facial and extremities dysmorphism (face: short columella, long philtrum, fingers: spatulate fingers, fetal pads), pigmented skin spots and abdominal patches, cryptorchidism, and psychomotor retardation. At 6 months of age, he presented with a first ischemic stroke revealed by hypotonic seizures during an upper respiratory infection. MRI showed recent ischemic lesions in the left MCA territory and intracranial arterial stenosis. He experienced a second ischemic stroke in the peri-operative period of cryptorchidy surgery (orchidopexia) at the age of 12 months associated with new ischemic lesions on MRI in the left MCA and ACA territories. Conventional angiography showed stenosis of terminal ICA bifurcations and deep neovessels suggestive of a bilateral MMA (Fig. 4). No abnormality was detected on trans-thoracic echocardiography, renal arteries Doppler examination, and ophthalmologic examination. Blood pressure was normal. Because of the syndromic presentation, a large chromosomal rearrangement was ruled out with the completion of a karyotype and a 730 K array analysis.

A cerebral revascularization using indirect techniques (bilateral multicraniostomy) was performed at the age of 17 months. Post-operative period was marked by a lack of transdural collateral development leading to a second targeted indirect surgery when 30-month-old. After cerebral revascularization, the patient presented a total of 6 ischemic strokes, 2 of them prompted by surgery (cryptorchidism) or crying, and 4 of them prompted by viral upper respiratory infections (associated respectively with VRS, HSV, and influenza B). At last examination (6 years old), he presented with asymmetrical bilateral motor deficit, severe oral dyspraxia and mental retardation. He was able to walk but could not speak. MRI performed during follow-up showed worsening of pre-existing arterial steno-occlusive lesions and involvement of posterior circulation on right and left PCAs (Fig. 4).

Proband M038 is a 52-year-old female born from healthy consanguineous parents (first cousins) originating from Turkey. Her familial history was marked by several cases of sudden death of unknown origin on both paternal and maternal sides. Her father died at 63 years after cardiac surgery. The patient developed high blood pressure at 25 years of age, requiring a triple antihypertensive therapy. At 43 of age, she presented a transient numbness of the right leg, followed two months later by a sudden episode of sensitivity disorder on the left hemibody. Cerebral MRI showed recent ischemic lesions in the superficial right MCA territory and in watershed areas of the left hemisphere and old ischemic lesions in the deep right MCA territory. Conventional angiography revealed an imaging pattern suggestive of MMA including steno-occlusive lesions of right intracranial ICA bifurcation associated with a deep right collateral network and an occlusion of the proximal part of left ACA (Fig. 4). Trans-thoracic echocardiography as CSF study didn’t detect any abnormality. Blood tests ruled out dyslipidemia, diabetes, inflammatory syndrome, but showed an isolated persistent IgG anticardiolipin antibody. Aorta CT angiography showed a 80% stenosis of the superior mesenteric artery ostium. A coronary computed tomography angiography was normal. A low dose Aspirin treatment was then started. The following months were marked by several transient episodes of left sensory-motor deficits revealing bilateral new punctiform ischemic lesions in the superficial right and left MCA territories. A treatment by Clopidogrel was then added to Aspirin. One year later, she suffered a transient left hemiparesis related to a hemodynamic transient ischemic attack (TIA) following a vasovagal syncope. Brain MRI and magnetic resonance angiography (MRA) showed a new ischemic lesion in the right ACA territory and a worsening of arterial steno-occlusions. Basal and acetazolamide brain perfusion SPECT with 99mTc-hexamethylpropyleneaminoxime (HMPAO) images showed a misery perfusion pattern in the right MCA and ACA territories. A cerebral revascularization surgery was then performed on the right side. This STA-MCA bypass was complicated by a cerebral hemorrhage related to a reperfusion syndrome associated with elevated blood pressure values leading to a persistent left hemiplegia.

During the following months, she presented with partial seizures which was treated par Levetiracetam and several hemodynamic TIAs. During the 7 following years of follow-up, she had no further cerebrovascular event.

Clinical and radiological features of the three mutated probands are summarized in Table 2, where are also listed the main characteristics observed in the *GUCY1A3* mutated MMA patients reported in literature [5, 11].

**Table 2:** Main clinical and neuroimaging features of the *NOS3* and *GUCY1A3* mutated probands reported in the present study and in literature

Patients	M084*	M035*	M038*	F1** (2 affected siblings)	F2** (2 affected siblings)	F3** (5 affected siblings)	M041***	M149***
Mutated gene	*NOS3*^*a*^	*NOS3*^*a*^	*GUCY1A3*^*b*^	*GUCY1A3*^*b*^	*GUCY1A3*^*b*^	*GUCY1A3*^*b*^	*GUCY1A3*^*b*^	*GUCY1A3*^*b*^
Variant description	HMZ variant	HMZ variant	HMZ variant	HMZ variant	HMZ variant	HMZ variant	Comp. HTZ variant	Comp.HT variant
	c.1502 + 1G > C	c.1942 T > C	c.1778G > A	c.1170delA	c.1045C > T	c.1086 + 1G > A	c.1258C > T AND c.1594G>T	c.334-335delGA AND c.1550G>A
	p.Ala502Trpfs*71	p.C648R	p.R593H	p.Glu391Lysfs*19	p.Arg349*	p.(?)	p.Arg420* AND p.Gly652*	p.Glu112fr AND p.Cys517Tyr
Gender	Female	Male	Female	Male/male	Female/female	4 males/1 female	Male	Female
Consanguinity	Yes	Yes	Yes	Yes	Yes	Yes	No	No
Moyamoya angiopathy	Yes	Yes	Yes	Yes (1/2)	Yes (1/2)	Yes (2/5)^c^	Yes	Yes
- Anterior circ. involvement	Yes	Yes	Yes	Yes	Yes	Yes (2/5)	Yes	Yes
- Posterior circ. involvement	Yes	Yes	No	Yes	Yes	Yes (1/5)	Yes	Not reported
MMA type according the first symptom	TIA	Ischemic stroke	TIA	Ischemic stroke	Ischemic stroke	Ischemic stroke (2/5)	Ischemic stroke	Ischemic stroke
Age at first MMA symptom	9m	6m	43y	3y	7m	2y/7m	18y	20m
MMA symptoms								
- TIA	Yes	No	Yes	No	No	Yes	Yes	No
- Ischemic stroke	Yes	Yes	Yes	Yes	Yes	Yes	No	Yes
- Hemorrhagic stroke	No	No	No	No	No	No	No	No
- Epilepsy	No	No	Yes	No	Yes	No	Yes	No
Other symptoms								
- Arterial hypertension	No	No	Yes	Yes (2/2)	Yes (2/2)	Not reported	Yes	Yes
- Systemic artery stenosis	No	No	Yes (SMA)	No	No	Not reported	Not reported	No
- Early-onset achalasia	No	No	No	Yes (2/2)	Yes (2/2)	Yes (5/5)	Yes	No
- Juvenile glaucoma	Yes	No	No	No	No	Not reported	Not reported	Not reported
- Raynaud and/or livedo	No	No	No	Yes (1/2)	Yes (1/2)	Not reported	Not reported	Not reported
- Other symptoms	No	IUGR, psychomotor delay, neonatal hypoglycemia, short stature, facial and extremities dysmorphism, pigmented skin spots and abdominal patches, cryptorchidism	No	Erectile dysfunction (1/2), low platelet count (1/2)			Gastroesophageal reflux, asthma, bilateral inguinal hernia, sudden death	Ascending aorta enlargement

Circ.:circulation; Comp.HTZ: compound heterozygous; HMZ: homozygous; IUGR: Intrauterine growth retardation; m: months; MMA: moyamoya angiopathy; NA: not applicable; SMA: superior mesenteric artery; TIA: transient ischemic attack; y: years

*Present study

**Hervé et al.

***Wallace et al.

^a^*NOS3* c.DNA variants are given for the transcript NM_000603.4

^b^*GUCY1A3* c.DNA variants are given for the transcripts NM_000856.5

^c^typical MMA in one sibling, unusual long arterial stenosis of the middle and anterior cerebral arteries in the other sibling

## Discussion

The analysis of six unrelated consanguineous MMA probands allowed us to identify in half of them pathogenic variants in the 2 major genes of the NO pathway. Two probands harbored two distinct homozygous variants in *NOS3*, the gene encoding the endothelial nitric oxide synthase (eNOS) which is the main source of NO in the vessel wall. The third one carried a homozygous mutation in *GUCY1A3*, the gene encoding the alpha1 subunit of the soluble guanylate cyclase (α1sGC) which is the major NO receptor in vascular smooth muscle cells. Functional analysis performed in endogenous circulating progenitor cells and transfected mutated cells showed that the two *NOS3* mutations caused either a complete absence or drastic reduction of eNOS. 3D in-silico structural analysis showed that the eNOS p.C648R substitution is expected to be destabilizing and to perturb proper folding of this region of the protein. The p.R593H substitution in the alpha1 subunit of sGC impacts the 3D structure in this region, the dynamics of the protein and its stability. Altogether, these data strongly suggest that all three mutations lead to an impairment of NO-sCG-cGMP signaling in the vascular wall and pinpoint the crucial role of NO signaling in MMA pathophysiology.

The role of the NO pathway in MMA pathogenesis was suspected following the previous description by our team of an early-onset MMA caused by homozygous truncating variants in the *GUCY1A3* gene in three unrelated families [5]. Two years later, two additional MMA single cases with compound heterozygous LOF variants of *GUCY1A3* were reported [11]. One of them carried, in addition to a nonsense variant, a missense p.C517T variant located in the catalytic domain of the protein.

**Fig. 5:**
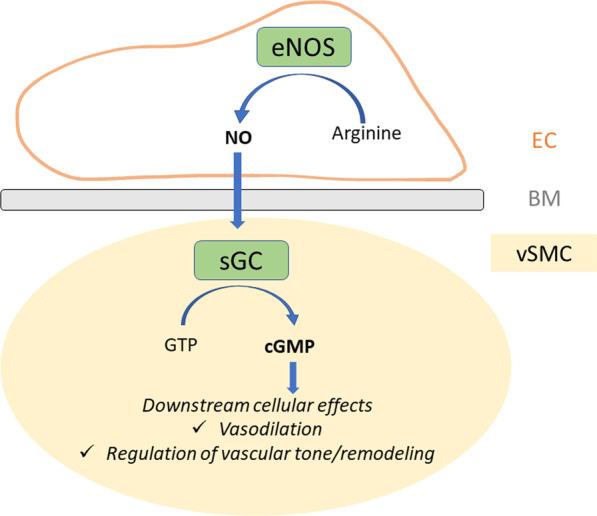
The NO-sGC-cGMP pathway in the vascular wall. NO is produced in the endothelium by endothelial NOS. It diffuses through the cellular membranes and reaches the soluble Guanylate Cyclase to activate cGMP production in the vascular smooth muscle cell. cGMP in turn activates downstream effectors involved in vasodilation, regulation of vascular tone and vascular remodeling. Legend: BM: basement membrane; EC: endothelial cell; eNOS: endothelial NO synthase; sGC: soluble Guanylate cyclase; cGMP: cyclic Guanosine Monophosphate; GTP: Guanosine Triphosphate; NO: nitric oxide; vSMC: vascular smooth muscle cell

The present study is the first report of MMA associated with *NOS3* LOF mutations. In addition, we describe herein for the first time a case of MMA carrying a rare bi-allelic missense variant of *GUCY1A3*. In total, half of the consanguineous probands that we investigated have LOF variants in one of the two major NO pathway genes, establishing the importance of a disruption of this pathway in MMA. NO is involved in the regulation of vascular tone and vascular remodeling [12]. In the vascular wall, NO is mainly produced by eNOS expressed in the endothelium. NO diffuses through membranes to activate the soluble guanylate cyclase (sGC) receptor expressed in adjacent vascular smooth muscle-cells [12, 13]. sGC produces in turn cyclic guanosine monophosphate (cGMP) and thus initiates downstream signaling through activation of cGMP-protein kinases, leading to the relaxation of vascular smooth muscle cells and vasodilation (Fig. 5) [14]. Beyond its role in vasodilation, NO is broadly involved in vascular wall maintenance and remodeling. Indeed NO acts as a negative regulator of smooth muscle cell (SMC) proliferation and migration in response to laminar shear stress [12, 15]. In atherosclerosis, and in post-injury arterial wall repair, it has been shown that NO pathway dysfunction contributes to migration of SMC from media to intima and to their proliferation [12, 16]. Pathological data from MMA arteries have shown an accumulation of intimal cells positive for smooth muscle alpha-actin (αSMA), an antigen usually expressed by SMC [17]. A pathophysiological hypothesis for MMA is that αSMA-positive cells accumulating in the neo-intima result from migration of proliferative SMC from media to intima. Altogether, these data strongly suggest that the alteration of the NO pathway in *NOS3* and *GUCY1A3* mutated MMA patients, might lead to abnormal vascular remodeling at sites where laminar flow is disrupted, such as arterial bifurcations, through an abnormal cell migration from media to intima [18]. However, the reasons underpinning the selectivity of the arterial lesions at the terminal ICA remains unknown.

In 6 out of the 9 *NOS3*/*GUCY1A3* mutated patients showing a MMA or a stenotic intracranial angiopathy reported to date (including ours), steno-occlusive arterial lesions involving PCA have been noticed (Table 2). This ratio is twice higher than the 30% of PCA involvement usually observed in MMA [19, 20]. PCA stenotic changes have been shown to negatively impact the prognosis of patients, as it is an independent risk factor predictive to cerebral infarction [21]. This high prevalence of posterior circulation involvement might contributes to the clinical severity of the *NOS3/GUCYA13* mutated patients who show a first ischemic event in early childhood in most cases (Table 2).

MMA is not the sole vascular condition associated with a disruption of NO signaling due to genetic mutations. Association studies have shown that heterozygous loss-of-function variants in *NOS3* and *GUCY1A3* genes were associated with an increased risk to develop hypertension and coronary heart disease [22]. In contrast, the same authors have shown that a common variant located in the *NOS3* promoter and known to enhance eNOS production was strongly associated with a decreased risk of hypertension and coronary artery disease. Some years before, Erdmann et al. reported a large family in which rare heterozygous mutations in *GUCY1A3* and *CCT7,* the latter encoding a scaffold protein which stabilizes sGC, were associated with premature coronary heart disease [23]. Interestingly, recent experimental data suggest that the NO pathway is functionally connected at the cellular level to *RNF213*, a giant ubiquitin ligase encoded by a major susceptibility gene in MMA [24, 25]. Altogether, these data strongly suggest that NO pathway disruption leads to the development of steno-occlusive lesions in various vascular beds, as observed in MMA (homozygous LOF mutations) or coronary heart disease (heterozygous variants). Of note, the family history of M038 proband is marked by several cases of coronary heart disease and unexplained sudden death. We suspect that these events could be related to the presence of the *GUCY1A3* p.R593H variant in a heterozygous state in M038 relatives on both maternal and paternal sides but this hypothesis needs to be further investigated to formally conclude.

Beyond their vascular phenotype, MMA patients harboring mutations in the NO pathway genes may show diverse extra-vascular symptoms. In addition to very-early onset MMA, M084 had a bilateral juvenile glaucoma, a rare subset of primary open-angle glaucoma. Open-angle glaucoma results from ocular hypertension, which is due to disequilibrium between the production and the clearance of aqueous humor in the eye’s anterior chamber. Several studies have shown that NO signaling is involved in regulation of the intraocular pressure through the control of the drainage of aqueous humor [26, 27]. Even though all three NOS enzymes are expressed in ocular tissues, eNOS seems to be preferentially involved. Experiments conducted in *NOS3* knock-out and knock-down mice have shown that eNOS is indeed involved in aqueous humor drainage, thus lowering the intraocular pressure [27]. In addition, several association studies showed a positive association between glaucoma and polymorphisms in the *NOS3* gene and in genes encoding caveolins 1 and 2, that modulate eNOS activity through regulation of their expression to the endothelial membrane [27]. Altogether, these data strongly suggest that the bilateral juvenile glaucoma of patient M084 is caused by complete loss of eNOS. Intriguingly, glaucoma has not been reported in M035. However, he was only 6-year-old at last examination, while diagnosis of glaucoma was done at age of 12 years in M084 proband. Moreover, the possibility of a residual eNOS activity in M035, who carries a *NOS3* homozygous missense variant and not a fully disruptive variant as the M084 proband does, could also explain this difference.

In addition to an early onset MMA, the M035 proband showed additional clinical features including a growth and psychomotor delay, dysmorphism, and multi-endocrine defects. We speculate that this complex phenotype, reported neither in M084 nor in *GUCY1A3* mutated patients, result of rare homozygous mutations involving other genes than *NOS3* in this consanguineous patient. Indeed, M035 proband carried 10 non-*NOS3* rare variants predicted to be deleterious, in contrast to M084 proband in whom the *NOS3* variant was the unique remaining variant after filtering (Table 1 and Additional file 3: Table S1).

Previously reported patients carrying homozygous LOF mutations in *GUCY1A3* showed, in addition to MMA, a severe achalasia which is absent in patient M038 [5, 11]. Another distinctive feature of M038 is the late-onset of MMA clinical manifestations contrasting with the very early onset in patients with disruptive biallelic mutations. Achalasia was also lacking in the proband reported by Wallace et al., patient who carried the p.C517T in addition to a nonsense variant [11]. Our current hypothesis is that the p.R593H missense variant found in M038 does not lead to a complete loss of sCG enzymatic activity and that the residual activity may prevent the development of achalasia. On the other hand, in the digestive tract, the production of NO by the neuronal NOS (nNOS, encoded by *NOS1* gene) explains that the patients mutated in *NOS3* do not suffer from achalasia.

In summary, we report here an early onset autosomal recessive MMA due to biallelic loss-of-function mutation in *NOS3*, the gene encoding the endothelial nitric oxide synthase, the main source of nitric oxide in the vascular wall. In addition, we showed that a bi-allelic missense mutation of *GUCY1A3* located in the catalytic domain of the protein may lead to MMA through its impact on the 3D structure of this region, the dynamics of the protein, local destabilization and the alteration of the flow of information transmission along the transducer module between the sensor module and the catalytic module. These data are important for genetic counseling regarding this severe condition. They improve our pathophysiological understanding of MMA emphasizing the role of NO signaling defects in MMA and opens avenues for therapeutic intervention [18].

## Supplementary Information

**Additional file 1:** Genealogical trees of to the 6 consanguineous MMA probands.

**Additional file 2:** 3D structural analysis of the functional consequences of the eNOS p.C648R variant and sGC alpha1 subunit p.R593H variant.

**Additional file 3:** Candidate homozygous variants identified in the 6 consanguineous probands.

**Additional file 4:** NOS3 homozygous splice variant in M084 and NOS3 homozygous missense variant in M035.

**Additional file 5:** Quantitative PCR results comparing relative expression of NOS3 mRNA in cells transfected with the wild type and with the M035 mutated cDNA.

## Data Availability

All data included in this study are available upon request by contact with the corresponding author.

## Abbreviations

ACA: Anterior cerebral artery
eNOS: Endothelial nitric oxide synthase
EPC: Endothelial progenitor cells
ES: Exome sequencing
GME: Greater Middle East
ICA: Internal carotid arteries
LOF: Loss-of-function
MAF: Minor allele frequency
MCA: Middle cerebral artery
MMA: Moyamoya angiopathy
MRI: Magnetic resonance imaging
NO: Nitric oxide
sGC: Soluble guanylate cyclase
